# Chemical Composition and In Vitro Cytotoxic and Antimicrobial Activities of the Essential Oil from Leaves of *Zanthoxylum monogynum* St. Hill (Rutaceae)

**DOI:** 10.3390/medicines4020031

**Published:** 2017-05-19

**Authors:** Fernanda B. da Silva, Nara O. dos Santos, Renata C. Pascon, Marcelo A. Vallim, Carlos R. Figueiredo, Roberto C. Campos Martins, Patricia Sartorelli

**Affiliations:** 1 Instituto de Pesquisas de Produtos Naturais Walter Mors, Universidade Federal do Rio de Janeiro, Rio de Janeiro 21941-902, RJ, Brazil; silvanandab@gmail.com (F.B.d.S.); roberto@nppn.ufrj.br (R.C.C.M.); 2 Instituto de Ciências Ambientais, Químicas e Farmacêuticas, Universidade Federal de São Paulo, Diadema 09972-270, SP, Brazil; nara.oshiro@gmail.com (N.O.d.S.); renata.pascon@gmail.com (R.C.P.); marcelo.vallim@gmail.com (M.A.V.); 3 Disciplina de Biologia Celular, Departamento de Micro, Imuno e Parasitologia, Universidade Federal de São Paulo, São Paulo 04023-062, SP, Brazil; rogernty@hotmail.com

**Keywords:** *Zanthoxylum monogynum*, Rutaceae, essential oil, cytotoxic activity, antimicrobial activity

## Abstract

**Background**: The *Zanthoxylum monogynum* species belongs to the family Rutaceae and is found in Southeast, Midwest, and Northeast Brazil. For this genus several biological activities have been described. **Methods**: The essential oil (EO) was obtained from the leaves of *Zanthoxylum monogynum* by hydro-distillation and was analyzed by gas chromatograph and gas chromatograph/mass spectrometry (GC and GC/MS). Also the EO of *Z. monogynum* was evaluated for in vitro cytotoxic activity against six tumor cell lines and for antimicrobial activity, performing disk diffusion and MIC assays with yeast and bacterial strains. **Results**: The chemical analysis afforded the identification of 18 components (99.0% of the EO). The major components were found to be citronellol (43.0%) and farnesol (32.0%). The *in vitro* cytotoxic activity against tumor cell lines, resulted in IC_50_ values ranging from 11–65 µg/mL against all tested cell lines. Antimicrobial activity of the essential oil was also tested and oil was effective, especially against Cryptococcus sp. yeast. All the tested yeast strains showed at least 90% growth inhibition. **Conclusions**: the essential oil from leaves of *Z. monogynum* has a different qualitative and quantitative composition when compared to the composition previously described. Also this EO has significant cytotoxic activity and moderate activity against *Cryptococcus* sp. and *Saccharomyces cereviseae* yeasts.

## 1. Introduction

The Rutaceae family has approximately 150 genera distributed worldwide in tropical, subtropical, and temperate regions. The *Zanthoxylum* genus comprises more than 200 species, being the most abundant genus in Rutaceae regarding to the number of species. In *Zanthoxylum* genus, it is common to find secondary metabolites such as alkaloids, lignans, coumarins, amides, flavonoids, and triterpenes [1,2]. 

In the *Zanthoxylum* genus, several biological activities—larvicidal [3], trypanocidal [4], anti-tumor [5], anti-psoriasis [6], and anti-inflammatory [7], among others—have been described. In Brazil, people of the state of Rondônia use the tea from leaves and roots of *Zanthoxylium* sp. to relieve toothache [8]. Additionally, the species *Z. simullans* and *Z. chalybeum* are used for the treatment of malaria [9,10]. 

Essential oils of various species of *Zanthoxylum* genus, such as the essential oil from branches of *Z. armantum*, which presented strong insecticidal activity against pests that attack stored products [11], have had their activities tested. Essential oil from the seeds of the same species has larvicidal activity against *Aedes aegypti, Anopheles stephensi,* and *Culex quinquefasciatus*, mosquitoes that are vectors of diseases [3].

The essential oil of *Z. gilletti* leaves showed larvicidal activity against *Anopheles gambiae*, the mosquito vector of malaria in Africa [12]. It has also been found that the essential oil *Z. alatum* exhibits antifungal and antibacterial activity [13], and the essential oil of *Z. simulans* exhibits anthelmintic activity [14]. The methanolic extract and the essential oil from fruits of *Zanthoxylum zanthoxyloides* and *Zanthoxylum leprieurii* presented anti-cancer and anti-microbial properties in vitro assays [15].

*Zanthoxylum monogynum* is popularly known as laranjeira-do-mato, tinguaciba-da-restinga, tinguaciba, limão-bravo, or limãozinho, occurring in Brazil in the states of Alagoas, Espírito Santo, Goiás, Minas Gerais, Pernambuco, Rio de Janeiro, and São Paulo [16]. The study of Maia and Andrade [17] in 2007, with leaves of *Z. monogynum* collected in different regions of Brazil, gave rise to two essential oils with different compositions and characteristics.

To date, there are no data in the literature about biological activities of the essential oil of *Z. monogynum*. The purpose of this study was (i) to give a chemical characterization of *Z. monogynum* essential oil, (ii) to investigate its cytotoxic activity in vitro against six tumor cell lines (B16F10, MCF-7, A2058, HeLa, HL-60 and T75), and (iii) to determine the antimicrobial effect of *Z. monogynum* essential oil against yeast (*Candida dubliniensis, Candida tropicalis, Candida glabrata, Candida parapsilosis, Candida krusei, Candida albicans, Cryptococcus grubii* (Serotype A), *Cryptococcus gattii* (Serotype B), *Cryptococcus gattii* (Serotype C), *Cryptococcus neoformans* (Serotype D), *Saccharomyces cerevisiae*), and Gram-positive and Gram-negative bacterial strains (*Escherichia coli*, *Serratia marcescens*, *Pseudomonas aeruginosa*, *Staphylococcus epidermidis*, and *Enterococcus faecalis*).

## 2. Materials and Methods

### 2.1. Plant Material

The plant material was collected in May 2013 in Itatiaia National Park, State of Rio de Janeiro. The species was identified by Dr. Marcelo Trovó (IB-UFRJ) and a voucher specimen is registered under the number MLO 601 (RB) in the herbarium (RBA) of the Federal University of Rio de Janeiro. The fresh leaves of *Z. monogynum* (185 g) were used to obtain the essential oil by a hydro-distillation process for 2 h at 80 °C; the residual water was removed through the addition of anhydrous sodium sulfate, which was then properly removed by a filtration procedure. An aliquot of the obtained essential oil was used for GC and GC/MS analysis.

### 2.2. Analysis of Essential Oil

The oil was analyzed by GC (Shimadzu®, Kyoto, Japan) on a gas chromatograph Shimadzu GC 2010 Plus (FID detector) (Shimadzu®, Kyoto, Japan) equipped with a DB-5 column (30 m × 0.25 mm × 0.25 μm) (Agilent® J&W GC Columns, Santa Clara, CA, USA). GC-MS analysis were performed on a mass spectrometer GCMS-QP2010 (Shimadzu®, Kyoto, Japan) at 70 eV with a DB-5 column (same as above). Oven temperature programmed from 60 °C to 246 °C at 3 °C min^−1^. For the injection (split 1:20), 5.0 μL of the essential oil were diluted in 500.0 μL of dichloromethane, and 1.0 μL of this diluted solution was injected. Identification of volatile constituents was made on the basis of their Kovats indexes (K.I.) and their mass spectra, which were compared with reference data [18].

### 2.3. Cell Lines

The following cell lines were obtained from the Ludwig Institute for Cancer Research (São Paulo, Brazil): human melanoma (A2058), human leukemia (HL-60), and human breast cancer (MCF-7). The human fibroblast T75, human cervical carcinoma (HeLa) cell, and the melanotic (B16F10) cell line, characterized by its low immunogenicity and moderate virulence, were obtained from the Experimental Oncology Unit (UNIFESP-Federal University of São Paulo, São Paulo, SP, Brazil). All tumorigenic cells were cultured in complete RPMI-1640 medium (Gibco, Grand Island, NY, USA) supplemented with 10% fetal bovine serum (FBS) (Gibco, Grand Island, NY, USA), 10 mM N-2-hydroxyethylpiperazine-N2 ethanesulphonic acid (HEPES; Sigma-Aldrich, St. Louis, MO, USA), 24 mM sodium bicarbonate, 40 mg/L gentamicin (Hipolabor, Minas Gerais, Brazil), at pH 7.2, and 37 °C in a humidified atmosphere with 5% CO_2_. Human T75 fibroblast was cultured in complete DEMEM medium (Gibco, Grand Island, NY, USA) supplemented with 10% of FBS and 40 mg/L gentamicin. All adherent cell lines were detached from the flask by using a PBS/EDTA solution (0.02% EDTA in PBS) and subcultured for 3–5 days until achieve 80% of max confluence or prior to be used in cytotoxic assays in vitro.

### 2.4. In Vitro Cytotoxic Activity

The essential oil from *Zanthoxylum monogynum* leaves was dissolved in dimethyl sulfoxide (DMSO) to a final concentration of 10.0 mg/mL, diluted in RPMI medium containing 10% fetal calf serum ranging from 100.0 to 0 µg/mL and incubated with 1 × 10^4^ cells in a 96-well plate (Corning, New York, NY, USA). After 18 h of incubation, cell viability was measured using the Cell Proliferation Kit I (MTT) (Sigma, Gillingham, UK), an MTT-based colorimetric assay [19]. Readings were made in a plate reader at 570 nm. All experiments were performed in triplicates.

### 2.5. Media, Antibiotics, and Growth Conditions

Yeasts were cultivated on agar plates containing Yeast Peptone Dextrose (YPD) (1% yeast extract, 2% peptone, 2% dextrose, and 2% agar) or RPMI 1640 (Sigma, Saint Louis, MO, USA). Gram-negative bacteria were grown in Luria Bertani (LB) (0.5% yeast extract, 1% tryptone, 1% NaCl, and 2% agar) and Gram-positive bacteria were tested in Brain Heart Infusion (BHI) (Himedia, Mumbai, India). Fluconazole (Sigma, Saint Louis, MO, USA) was used as positive controls for yeast and chloramphenicol (Sigma, Saint Louis, MO, USA) was the positive control for bacteria. Crude essential oil from *Z. monogynum* was diluted in DMSO or saline (0.9%) plus Tween 80 (0.5%) and spotted on 5 mm sterile filter paper [20].

### 2.6. Microorganisms Strain

The bacteria and yeast species used in this work are described in Table 1. These strains where obtained from CBMAI (Coleção Brasileira de Micro-organismos de Ambiente e Indústria) and ATCC (American Type Culture Collection).

### 2.7. Disk Diffusion Assay

To assess the antimicrobial activity of *Z. monogynum* essential oil, the disk diffusion method described by the Clinical and Laboratory Standards Institute (CLSI, OPAS M2-A8) was used with some modification. Thin agar plates with 10 mL of either YPD (yeast) or LB (Gram-negative) or BHI (Gram-positive) media supplemented with 2% agar were covered with 10 mL of soft agar medium (YPD, LB, or BHI plus 1% agar) containing 100 μL of each fresh culture (adjusted to OD_600_ = 0.3) of yeast or bacteria grown for 12–16 h in 3 mL of liquid media incubated at 30 °C/37 °C with aeration (150 rpm). After the agar was solidified, 5 mm filter paper disks sterilized and impregnated with 20 μL of crude essential oil diluted in DMSO were placed on top of agar plates and incubated at 30 °C/37 °C for 24 or 48 h depending on the microorganism. Fluconazole (1 mg) and chloramphenicol (200 μg) impregnated disks were used as positive controls for yeast and bacteria, respectively. Negative control was prepared by paper disks impregnating with DMSO at the same concentration employed to dilute the essential oil. All tests were performed in duplicates. Tested strains were selected based on their clinical importance and previous results obtained by our research group [20].

### 2.8. Minimum Inhibitory Concentration

Microdilution tests were conducted according to the Clinical and Laboratory Standards Institute (CLSI), OPAS1 M27-A2 for yeasts and OPAS M7-A6 for bacteria according to literature [20] with slight modifications. Briefly, MIC values were determined using micro titer plates (96 wells) in a total volume of 100.0 µL. Microorganisms were cultured in test tubes filled with 3 mL of medium (RPMI 1640 for yeast and BHI for bacteria) overnight at 30 °C in a rotary shaker (150 rpm). The cultures were diluted and adjusted to 1–2 × 10^2^ CFU/mL using a McFarland´s scale. The CFU counts and viability was confirmed by viability counts on YPD and BHI plates (100.0 µL of diluted cells). Crude essential oil and reference standards were serial diluted (two-fold) and added to each well. A negative control containing medium only and growth control containing cell, DMSO (10.0 µL), or saline (10.0 µL) and Tween 80 were included as negative and positive controls, respectively. Depending on the microorganism, the micro titer plates were then incubated at 30 °C for 24 or 48 h. Microorganism growth was determined by reading the absorbance at 530 nm in a plate reader (Logen, MT-960, BioTek Instruments, Winooski, VT, USA), and the minimum inhibitory concentration was considered the lowest concentration at which at least 80% of growth was inhibited. All tests were performed in triplicate.

## 3. Results and Discussion

### 3.1. Chemical Composition of the Essential Oil from Z. monogynum Leaves 

The hydrodistillation of the 185.0 g of leaves resulted in 1.1 g of essential oil. Analysis by gas chromatography with flame ionization detector (GC/FID) and chromatography coupled to mass spectrometry (GC/MS) and calculation of Kovats indexes and comparison with the literature data [18] allowed for the identification of 18 compounds (Table 2), which represents 99.06% of the oil. The major compound is citronellol (43.03%), a monoterpenoid that is followed by the sesquiterpenoid farnesol (31.96%). Another monoterpenoid, citronellal (9.57%), was identified followed by D-germacrene (4.52%), β-pinene (2.02%), δ-elemene (1.38%), and (Z)-β-farnesene (1.20%). Together, these seven compounds correspond to 93.68% of the oil composition.

In the study performed by Maia and Andrade in 2007 [17] with the essential oil of *Z. monogynum,* from different regions, the composition of each of them is described: In one of the major compounds was the oils citronellal (57.1%), limonene (13.8%), citronellyl acetate (13.1%), and citronellol (6.2%). In the other, the main compounds were 2-undecanone (70.2%) and citronellal (22.5%). It is believed that this difference in composition might be related to the use of leaves from two chemotypes of *Zanthoxylum monogynum*. Additionally, some factors can affect this difference in composition such as geographical locations, environment, and stage of maturity [21]. 

### 3.2. Citotoxic Activity

The essential oil from the leaves of *Z. monogynum* was evaluated for its cytotoxic activity in vitro against six tumor cell lines (B16F10, MCF-7, A2058, HeLa, HL-60, and T75). The crude oil displayed activity against all cell lines tested, with IC_50_ values ranging from 11.0 to 65.0 µg/mL, HL60 being the most sensitive cell line to the crude oil, with an IC_50_ of 11.0 μg/mL, followed by A2058 with an IC_50_ value of 34.0. Other tumor cell lines BF16F10, MCF-7, and HeLa were less sensitive with IC_50_ values of 60.0, 65.0, and 62.0 respectively (Table 3). 

In a previous study concerning the cytotoxic activity of essential oil of another species of *Zathoxylum* (*Z. fagara*), whose major constituent was citronellol (26%), was evaluated against MDAMB-231 (human breast adenocarcinoma), Hs 578T (human breast ductal carcinoma), and 5637 (human primary bladdercarcinoma) cells. Neither the essential oil itself nor its major compounds exhibited notable cytotoxic activity against these cell lines [22].

However, the effects of essential oils have been investigated against diverse tumor cells such as glioblastoma, melanoma, leukemia, and oral cancers, as well as colon, kidney, and others [23]. Additionally essential oils from *Casearia sylvestris* [24] and *Lippia alba* [25] have been described as cytotoxic against melanoma murine (BF16F10). Furthermore, several essential oils could have anticancer effects, as there is a relationship between the production of reactive oxygen species and the origin of oxidation and inflammation that can lead to cancer [23].

The results presented here, where the essential oil from *Z. monogynum* showed similar or lower IC_50_ values (in the case of MCF-7 and HL-60 cell lines) in comparison to standard drugs (cisplatin and paclitaxel) against the tested cells, suggest that it could be further explored as a source for compounds with antitumor activity. Thus, the essential oil from *Z. monogynum* could be considered as a source of antitumor compounds.

### 3.3. Antimicrobial Activity

Regarding antimicrobial activity, initially, disk diffusion assays were conducted with the yeasts and bacterial strains listed in Table 1 in order to screen for antimicrobial activity. No bacterial strains were sensitive to essential oil from *Z. monogynum*, but the crude oil was sensitive to yeast, especially *Cryptococcus* sp. (Supplementary Table S1). Since no biological activity was detected for bacterial strain in this qualitative assay, no further experiments were conducted with these microorganisms. However, the MIC test applied to the yeast species listed in Table 1 confirmed that all yeast strains were sensitive to essential oil at concentrations ranging from 0.75 (*S. cerevisae*) to 6.0 mg/mL (*Candida* sp.) with 90% growth inhibition (Table 4).

Several essential oils and their major constituents from medicinal plants have been reported to possess a wide range of bacterial and fungal inhibitory potentials [26,27]. Additionally, citronellol has been described as active against *Candida tropicalis* [28]. However, the selectivity index (SI_50_ = IC_50_/MIC) estimated for this essential oil is low, meaning that it may have low selective toxicity. In the future, this matter could be overcome by chemical modifications in the inhibitor.

## 4. Conclusions

According to the results, the essential oil from leaves of *Z. monogynum* has a different qualitative and quantitative composition when compared to the composition previously described, showing sesquiterpenes citronellol and farnesol as major constituents. Lower IC_50_ values for the tested tumor cell lines proved that this EO has significant cytotoxic activity. EO from *Z. monogynum* has also proven to exhibit moderate activity against *Cryptococcus* sp. and *Saccharomyces cereviseae* yeasts, but not significant activity against *Candida* bacterial strains. Analysis of the EO composition suggests that the major compounds might not be responsible for the observed activities as citronellol itself was previously tested against *Candida tropicalis* and showed to be potent against this bacterium.

## Figures and Tables

**Table 1:** Target strains used for antimicrobial activity assays.

Species	Designation
**Yeast**	
*Candida dubliniensis*	ATCC 7978
*Candida tropicalis*	ATCC 13803
*Candida glabrata*	ATCC 90030
*Candida parapsilosis*	Clinical isolate 68
*Candida krusei*	Clinical isolate 9602
*Candida albicans*	CBMAI 560
*Cryptococcus grubii* (A)	ATCC 208821
*Cryptococcus gattii* (B)	ATCC MYA-4563
*Cryptococcus gattii* (C)	ATCC MYA-4560
*Cryptococcus neoformans* (D)	ATCC MYA-4567
*Saccharomyces cerevisiae*	ATCC 201389
**Bacteria**	
*Escherichia coli*	-
*Serratia marcescens*	CBMAI 469
*Pseudomonas aeruginosa*	CBMAI 602
*Staphylococcus epidermidis*	CBMAI 604
*Enterococcus faecalis*	-

-, not known.

**Table 2:** Chemical composition of the essential oil of *Z. monogynum*.

KI	RT (min)	Compound	Molecular Weight	%
855	2.825	2-hexenal	98	0.20
939	4.117	α-pinene	136	0.99
971	4.967	β-tujeno	136	0.41
979	5.100	β-pinene	136	2.02
990	5.350	β-myrcene	136	0.30
1002	5.817	α-phellandrene	136	0.17
1029	6.442	D-limonene	136	0.90
1037	6.950	α-ocimene	136	0.42
1082	8.708	β-linalool	154	0.52
1153	10.600	citronellal	154	9.57
1225	13.550	citronellol	156	43.03
1257	14.663	methyl citronelate	184	0.45
1338	17.467	δ-elemene	204	1.38
1352	18.258	citronellol acetate	198	0.25
1442	22.158	(*Z*)-β-farnesene	204	1.20
1481	22.975	D-germacrene	204	4.52
1436	25.775	γ-elemene	204	0.77
1743	31.717	farnesol	222	31.96
		**Total**		99.06

**Table 3:** IC_50_ (μg/mL) values to essential oil from *Z. monogynum* leaves and to positive controls (cisplatin and paclitaxel) against tumor cell lines.

Cell Lines	IC_50_ (µg/mL)		
	Essential Oil	Cisplatin	Paclitaxel
B16F10	60.0 ± 8.9	52.8 ± 4.50	-
A2058	34.0 ± 8	43.1 ± 3.6	-
MCF-7	65.7 ± 2.8	-	171.5 ± 16.39
HeLa	62 ± 5.9	20.3 ± 1.20	-
HL-60	11.0 ± 1	20.9 ± 1.50	-
T75	60 ± 4	nd	nd

nd, not determined; -, not evaluated.

**Table 4:** Minimum inhibitory concentrations (MICs) for *Z. monogynum* essential oil and positive control (fluconazole) both expressed in mg/mL. Numbers in parenthesis represent the average percentage inhibition for each MIC followed by the standard deviation.

Species	OEZM (mg/mL)	Fluconazol (mg/mL)
*Candida albicans*	3.0 (99.44% ± 0.03)	0.025 (98.41% ± 0.21)
*C. dubliniensis*	3.0 (95.65% ± 4.05)	0.013 (99.47% ± 0.07)
*C. tropicalis*	3.0 (99.23% ± 0.16)	0.05 (93.59% ± 0.46)
*C. glabrata*	6.0 (97.78% ± 0.59)	0.05 (94.49% ± 8.22)
*C. parapsilosis*	6.0 (98.78% ± 0.21)	0.025 (86.27% ± 3.27)
*C. krusei*	6.0 (98.84% ± 0.78)	0.05 (85.34% ± 6.67)
*Cryptococcus neoformans* var.* grubii *(sorotipo A)	1.5 (92.12% ± 0.39)	0.025 (92.82% ± 0.38)
*C. neoformans* var. *gattii *(sorotipo B)	1.5 (95.26% ± 0.67)	0.013 (98.84% ± 0.05)
*C. neoformans* var. *gattii *(sorotipo C)	1.5 (92.47% ± 0.76)	0.013 (98.05% ± 0.12))
*C. neoformans* var.* neoformans* (sorotipo D)	1.5 (91.96% ± 0.62)	0.025 (99.54% ± 0.65)
*Saccharomyces cerevisiae*	0.75 (94.54% ± 0.90)	0.025 (91.94% ± 0.04)

## Supplementary Materials

The following are available online at www.mdpi.com/2305-6320/4/2/31/s1, Table S1: Inhibition zone (IZ) expressed in centimeters (cm) produced by the positive control (1 mg of chloranphenicol for bacteria and 200 µg of fluconazole for yeast strains) and OEZM.

## Abbreviations

The following abbreviations are used in this manuscript:

GC	gas chromatograph
GC/MS	gas chromatograph/ mass spectrometry
K.I.	Kovats Index
R.T.	Retention Time
OEZM	essential oil of Zanthoxylum monogynum
MIC	Minimum Inhibitory Concentration
IB-UFRJ	Institute of Biology—Federal University of Rio de Janeiro
DMSO	dimethyl sulfoxide
