# Supplementary material for: Chemical Composition and In Vitro Cytotoxic and Antimicrobial Activities of the Essential Oil from Leaves of Zanthoxylum monogynum St. Hill (Rutaceae)
Source: Medicines (Basel). 2017 May 19;4(2):31. doi: 10.3390/medicines4020031 (PMC5590067; doi:10.3390/medicines4020031)
Supplement: Supplementary file 1 [file medicines-04-00031-s001.pdf]

Supplementary Materials: Chemical Composition and In Vitro Cytotoxic and Antimicrobial Activities of the Essential Oil from Leaves of Zanthoxylum monogynum St. Hill (Rutaceae)

Fernanda B. da Silva, Nara O. dos Santos, Renata C. Pascon, Marcelo A. Vallim, Carlos R. Figueiredo, Roberto C. Campos Martins and Patricia Sartorelli

Table S1. Inhibition zone (IZ) expressed in centimeters (cm) produced by the positive control (1 mg of chloranphenicol for bacteria and 200 µg of fluconazole for yeast strains) and OEZM.

Microbial species	Positive Control IZ (cm)	OEZM IZ (cm)
E. coli	1,9	0 ± 0,00
S. marcescens	1,3	0 ± 0,00
P. aeruginosa	0,9	0 ± 0,00
E. faecalis	1,4	0 ± 0,00
S. epidermidis	1,4	0 ± 0,00
C. albicans	2,2	0,85 ± 0,07
C. dubliniensis	2,0	0,45 ± 0,07
C. tropicalis	1,9	0,45 ± 0,07
C. glabrata	0,7	0,45 ± 0,07
C. parapsilosis	1,8	0,5 ± 0,14
C. krusei	0,9	0,45 ± 0,07
C. neoformans (A)	1,0	2,4 ± 0,00
C. gattii (B)	1,2	1,95 ± 0,07
C. gattii (C)	0,5	0,5 ± 0,14
C. neoformans (D)	1,5	2,2 ± 0,28
S. cerevisiae	0,6	0,45 ± 0,07

The numbers reflect average and standard deviation of two replicates.
